# Visibility of Pulmonary Valve and Pulmonary Regurgitation on Intracardiac Echocardiography in Adult Patients with Tetralogy of Fallot

**DOI:** 10.3390/jcdd10010024

**Published:** 2023-01-07

**Authors:** Ichiro Sakamoto, Kenichiro Yamamura, Ayako Ishikita, Kisho Ohtani, Shintaro Umemoto, Hidetaka Kaku, Yuzo Yamasaki, Kohtaro Abe, Tomomi Ide, Hiroyuki Tsutsui

**Affiliations:** 1 Department of Cardiovascular Medicine, Graduate School of Medical Sciences, Kyushu University, Fukuoka 812-8582, Japan; 2 Department of Pediatrics, Graduate School of Medical Sciences, Kyushu University, Fukuoka 812-8582, Japan; 3 Department of Cardiovascular Intensive Care, Fukuoka Children’s Hospital, Fukuoka 813-0017, Japan; 4 Department of Cardiovascular Internal Medicine, National Hospital Organization, Kyushu Medical Center, Fukuoka 810-8563, Japan; 5 Department of Clinical Radiology, Graduate School of Medical Sciences, Kyushu University, Fukuoka 812-8582, Japan

**Keywords:** intracardiac echocardiography, pulmonary valve, pulmonary regurgitation, tetralogy of Fallot

## Abstract

Pulmonary regurgitation (PR) is a risk factor for sudden cardiac death in adult patients with repaired tetralogy of Fallot (TOF). However, transthoracic echocardiography (TTE) cannot fully visualize the pulmonary valve (PV) and PR. We investigated whether intracardiac echocardiography (ICE) could visualize the PV and PR better than TTE. Thirty adult patients with TOF (mean age 33 ± 15 years) scheduled for cardiac catheterization underwent ICE. The visualization of PV and the severity of PR were classified into three grades. ICE depicted the PV better than TTE (ICE vs. TTE: not visualized, partially visualized, and fully visualized: *n* = 1 [3%], *n* = 13 [43%], and *n* = 16 [53%] vs. *n* = 14 [47%], *n* = 13 [43%], and *n* = 3 [10%], *p* < 0.001). Especially in patients after pulmonary valve replacement (PVR), the PV was more fully visualized by ICE. The assessment of PR by TTE underestimated the severity of PR in comparison to cardiac magnetic resonance imaging (MRI) (severe PR: 8 [28%] vs. 22 [76%], *p* = 0.004), while there was no discrepancy between the results of ICE and MRI (21 [72%] vs. 22 [76%], *p* = 1.000). In comparison to TTE, ICE can safely provide better visualization of the PV and PR in adults with TOF, especially in patients who have undergone PVR.

## 1. Introduction

The number of adult patients with congenital heart disease is increasing worldwide [1]. Significant advances in childhood surgical repair have enabled most patients with tetralogy of Fallot (TOF) to reach adulthood. However, sudden cardiac death and heart failure are major late complications in adult patients with repaired TOF [2]. Pulmonary regurgitation (PR) is a known risk factor for these lethal complications [3]. Surgical or percutaneous pulmonary valve replacement (PVR) has recently been performed for PR with right ventricular dilatation and dysfunction. However, PVR using bioprosthetic valves may require further treatment due to the prosthetic valve failure [4]. The evaluation of pulmonary valve stenosis or regurgitation is necessary to perform retreatment at appropriate timing. The assessment of bioprosthetic valves using transthoracic echocardiography (TTE) is especially challenging in comparison to the native valve. Visualization of the pulmonary valve (PV) is often difficult, partly due to the limited acoustic window of TTE [5]. Furthermore, transesophageal echocardiography (TEE) cannot provide ideal visualization of the PV because of the distance from the probe to the PV. Diagnostic cardiac catheterization requires contrast agents and a detailed assessment of the PV and PR is often difficult because the catheter passes the PV. In addition, there is a rare but non-negligible risk of bioprosthetic pulmonary valve injury in patients after PVR. Cardiac magnetic resonance imaging (MRI) is a gold standard for the quantitative assessment of PR; however, morphological assessment is not sufficient due to low spatial resolution.

Intracardiac echocardiography (ICE) is usually used to guide invasive procedures, such as myocardial biopsy [6], device closure of secundum atrial septal defects [7] and puncture of the atrial septum before pulmonary vein isolation [8]. Conventional cardiac catheterization is needed to assess the hemodynamics of adult patients with TOF. However, it is not very useful for the morphological assessment of PV. Theoretically, introducing the ICE probe into the right ventricular outflow tract would provide the best visualization of the PV because there is no structural barrier. A sufficient morphological assessment of the PV enables a better understanding of the mechanism and severity of the PR, which is essential to determine the appropriate timing and treatment option for reintervention.

The aim of the present study is to investigate whether ICE could visualize the PV and PR better than TTE in adults with TOF.

## 2. Materials and Methods

### 2.1. Patients Population

Adult patients with TOF or pulmonary atresia with a ventricular septal defect (PA/VSD) who were scheduled for diagnostic cardiac catheterization were enrolled in Kyushu University Hospital between July 2013 and September 2017. Patients who were scheduled for cardiac intervention were excluded from the study.

### 2.2. Transthoracic Echocardiography (TTE)

In all cases TTE was performed using an iE33/EPIQ 7 (Philips Health care, Andover, MA, USA) or Vivid 7 (GE Medical Systems, Milwaukee, WI, USA). The PV morphology was assessed on the parasternal short axis view. The severity of PR was evaluated according to the recent American Society of Echocardiography (ASE) guidelines [8].

### 2.3. Intracardiac Echocardiography (ICE)

**Figure 1:**
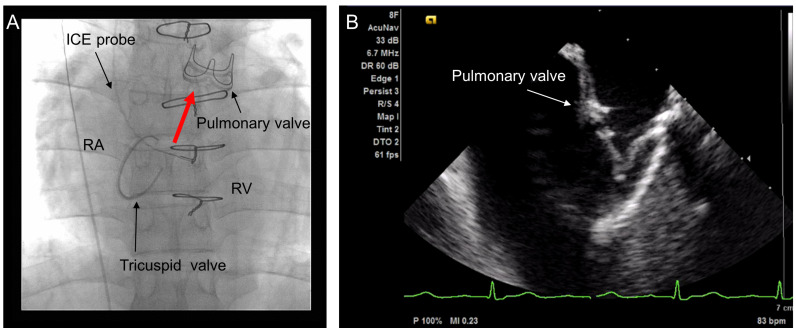
(**A**) Cine angiogram after pulmonary valve replacement (PVR) and (**B**) intracardiac echocardiography (ICE) from the internal jugular vein. A 33-year-old man diagnosed with tetralogy of Fallot underwent intracardiac repair at 6 years of age and PVR with tricuspid ring annuloplasty at 32 years of age. The ICE probe was inserted from the internal jugular vein. The red arrow shows the echo beam. ICE shows excellent visualization of the prosthetic pulmonary valve. RA, right atrium; RV, right ventricle.

ICE was performed on all patients after the diagnostic cardiac catheterization. For cardiac catheterization and ICE sedation was performed with pentazocine (7.5 to 15 mg) and hydroxyzine (12.5 to 25 mg). All patients underwent right heart catheterization from the internal jugular vein. They received 3000 units intravenous heparin after sheath insertion. The ICE catheter was inserted from the internal jugular vein using a 9-Fr or 10-Fr 11 cm sheath. An 8-Fr AcuNav^TM^ Ultrasound Catheter (Biosense Webster Inc., Irvine, CA, USA) or the 9-Fr ViewFlex^TM^ Xtra ICE Catheter (St. Jude Medical, Atrial Fibrillation Division, Inc., Minnesota, USA) were used. Both of them were only 2D-capable. After inserting the ICE catheter into the RA, it was advanced to the RV under fluoroscopy in a similar manner to the insertion of a Swan-Ganz catheter. With anterior deflection at the height of the tricuspid valve, the ICE catheter could easily enter into the RV. The PV was visualized from the right ventricle without difficulty (Figure 1). All the manipulation in ICE was performed by a single expert interventional cardiologist (I.S.).

### 2.4. Assessment of the PV and PR

**Figure 2:**
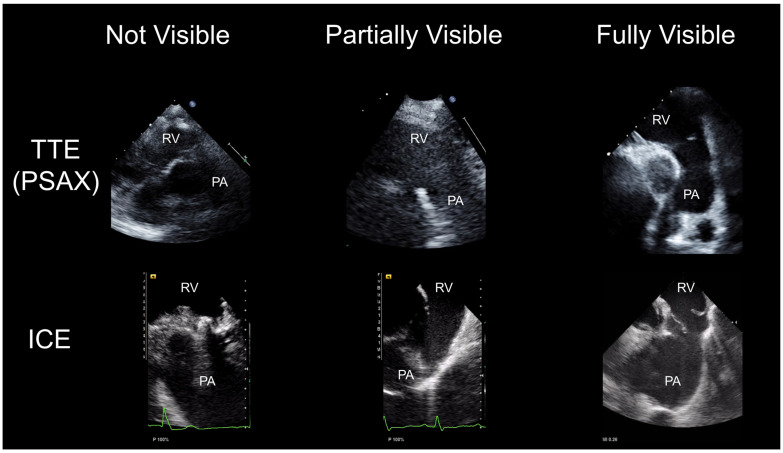
Echocardiographic assessment of PV using TTE and ICE. Representative imaging of the “not visible”, “partially visible” and “fully visible” classifications by TTE and ICE. The upper and lower images are not of the same patients. TTE, transthoracic echocardiography; ICE, intracardiac echocardiography; RV, right ventricle; PA, pulmonary artery; PSAX, parasternal short axis.

Morphological assessments of the PV using TTE and ICE were performed by two cardiologists (S.U. and K.H.) on the same day. The visualization of the PV in TTE and ICE was classified into three grades; not visible, partially visible, and fully visible (Figure 2). Cases in which only part of the pulmonary valve annulus was observed were defined as “partially visible.” Cases in which the PV annulus and leaflet were entirely observed were defined as “fully visible”. The severity of PR was classified into three grades: mild, moderate, and severe, based on the ASE guidelines [9]. We mainly evaluated the severity of PR using a visual assessment with 2D and color Doppler. When there was discrepancy in the evaluations of the two cardiologists, a reevaluation was performed and the grade was decided after discussion.

### 2.5. Cardiac Magnetic Resonance Imaging

To evaluate the size and function of the RV and left ventricle (LV), cardiac MRI was performed before cardiac catheterization. All patients underwent 3-Tesla MRI (Achieva 3.0T TX or Ingenia 3.0T CX; Philips Healthcare, Best, The Netherlands), which was equipped with dual-source parallel radiofrequency transmission, 32-channel phased-array torso coils used for radiofrequency reception and a four-lead vector cardiogram used for cardiac gating. RV volumes were measured with axial cine MRI images, as reported in the past [10]. Phase-contrast velocity mapping with a flow-sensitive, gradient-echo sequence was performed in the main pulmonary artery. PR was graded as mild with pulmonary regurgitation fraction (PRF) < 20%, moderate between 20% and 35%, and severe > 35%. Cardiac MRI images were analyzed semi-automatically, followed by manual correction using a workstation (IntelliSpace Portal, Philips Healthcare, Best, The Netherlands).

### 2.6. Data Analysis

We used Fisher’s exact test to analyze differences in categorical variables between the two groups. Cohen’s kappa coefficient was used to measure the agreement between the two observers. *p* values of <0.05 were considered statistically significant. All statistical analyses were performed using JMP Pro 14 (SAS Institute Inc., Cary, NC, USA).

## 3. Results

### 3.1. Patient Characteristics

A total of 30 adult patients with TOF or PA/VSD were included in the present study (Table 1). The mean age of the patients was 33 ± 14 years, and 18 (60%) patients were female. Five patients were diagnosed with PA/VSD and all of them underwent Rastelli type operations. Four of them underwent PVR (Table 1). Only 1 of the 30 patients had not undergone previous cardiac surgery. Eight patients underwent PVR with a bioprosthetic valve (CEP, *n* = 6; SJM Epic, *n* = 1; Hancock II, *n* = 1). Twenty-seven patients (90%) were in sinus rhythm during TTE and ICE. One patient had undergone implantation of a permanent pacemaker.

The hemodynamic data obtained by cardiac catheterization are shown in Table 2. There were 5 patients (17%) with moderate pulmonary stenosis (systolic RV pressure − systolic PA pressure > 30 mm Hg). No patients showed elevated pulmonary artery pressure or pulmonary capillary wedge pressure.

The conventional assessment by TTE is shown in Table 3. There were 9 patients (30%) with severe PR, 4 patients (13%) with moderate PR and 26 patients (26%) with mild PR. The left ventricular ejection fraction was normal in all patients.

### 3.2. Cardiac Magnetic Resonance Imaging

Cardiac MRI was performed in 29 patients (except for the one who had permanent pacemaker implantation using a non-MRI-conditional epicardial lead). There were 18 patients (62%) with severe PR and 5 patients (17%) with moderate PR. The pulmonary regurgitation fraction (PRF) was 37 ± 21%. The right ventricle was dilated and the right ventricular ejection fraction was mildly impaired (Table 4).

### 3.3. Visualization of the PV

**Figure 3:**
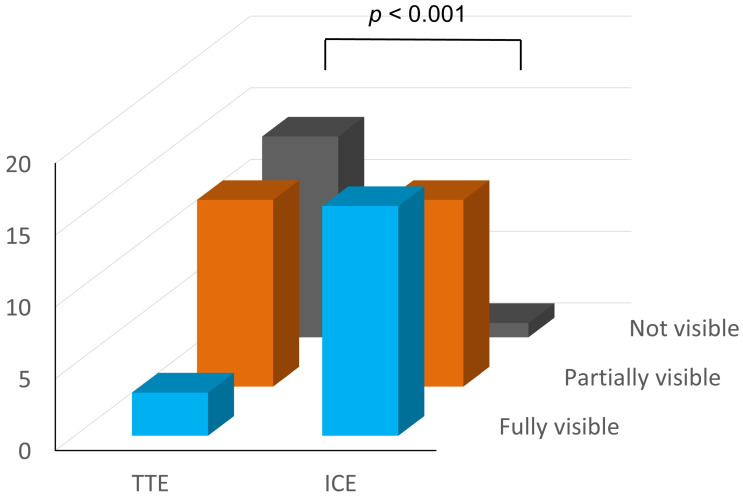
Visualization of the PV by TTE and ICE. The PV was not visible on TTE in nearly half of the patients. However, the PV was fully visible or partially visible on ICE in most cases (TTE vs. ICE, *p* < 0.001, Fisher’s exact test). TTE, transthoracic echocardiography; ICE, intracardiac echocardiography.

ICE depicted the PV better than TTE (ICE vs. TTE: not visualized, partially visualized, and fully visualized: *n* = 1 [3%], *n* = 13 [43%], and *n* = 16 [53%] vs. *n* = 14 [47%], *n* = 13 [43%], and *n* = 3 [10%], *p* < 0.001) (Figure 3). ICE was especially superior in the visualization of the bioprosthetic pulmonary valve. No bioprosthetic pulmonary valve was fully visualized by TTE. In contrast, 5 (63%) of the 8 bioprosthetic pulmonary valves were fully visualized by ICE (*p* = 0.006). Cohen’s kappa coefficient showed very good agreement between the assessments of the two observers (0.883 and 0.881 for TTE and ICE, respectively).

### 3.4. Accuracy in the Assessment of PR

**Figure 4:**
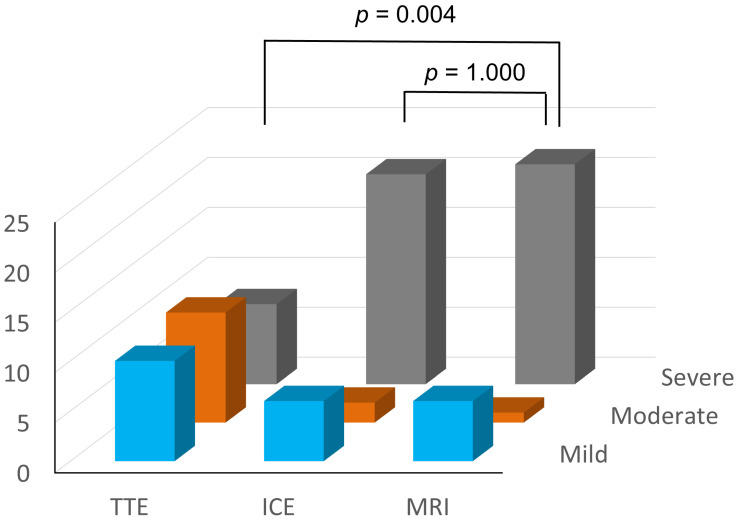
Comparison of assessment of the severity of PR by TTE, ICE and MRI. TTE underestimated the severity of PR in comparison to ICE and MRI (TTE vs. MRI *p* = 0.004, ICE vs. MRI *p* = 1.000, Fisher’s exact test). TTE, transthoracic echocardiography; ICE, intracardiac echocardiography; MRI, magnetic resonance imaging.

The severity of the PR was assessed in 29 patients who underwent cardiac MRI (Figure 4). TTE underestimated the severity of PR in comparison to cardiac MRI (severe PR: 8 [28%] vs. 22 [76%], *p* = 0.004). On the other hand, the number of patients who were diagnosed as having severe PR was not significantly different between ICE and MRI (21 [72%] vs. 22 [76%], *p* = 1.000).

### 3.5. Safety of ICE

We performed right heart cardiac catheterization and ICE from the internal jugular vein in all cases. The average time required to perform ICE assessment was 5 min. No procedure-related adverse events (death, thrombus formation, cardiac tamponade, lethal arrhythmia, and bleeding) were observed in the present study. Premature ventricular contraction and its short-run were occasionally observed during the manipulation of ICE. However, there were no cases that experienced sustained ventricular tachycardia or hemodynamic deterioration.

## 4. Discussion

This is the first study to demonstrate the superiority of ICE in the assessment of PV morphology and the severity of PR in comparison to TTE. The native PV is relatively thin and has highly pliable leaflets, which makes its entire visualization by TTE difficult [9]. In adult patients with TOF, the right ventricular outflow tract and PV were often not visualized by TTE due to limited echo window in the post-operative state [5]. Using ICE, most PVs could be well visualized in adult patients with TOF. Recently, various techniques for valve-sparing or valve reconstruction surgeries have been utilized to reduce the risk of future PR [11]. There is still controversy about the indications for and choice of these techniques. A detailed morphological evaluation with ICE can provide useful information as feedback for the cardiac surgeons to further improve these procedures.

Several invasive and non-invasive modalities are available for assessing the anatomy of the PV. Cardiac MRI is useful for volume and flow measurement, while it is not the best option for morphological assessment of the PV. CT provides very good spatial resolution. However, the time resolution is inferior to TTE and ICE. The artifact caused by the sewing ring after PVR is also an obstacle to the clear depiction of the valve. 3D TEE is superior to TTE in the assessment of the pulmonary valve. However, the PV is the most anteriorly and superiorly located valve, and it is often challenging to visualize the PV because of interference from other structures, and its far-field location. It is important to choose the best modality for the assessment of the PV function and ICE could be a reliable diagnostic tool, especially when the patient is scheduled for cardiac catheterization. Although we have not performed a quantitative evaluation of PR with ICE, multiple Doppler parameters may improve the quality of the evaluation of PR. 

Surgical PVR for severe PR has been performed in recent years [12]. However, over the long term, bioprosthetic pulmonary valves may deteriorate. There have been several reports of late prosthetic valve failure after PVR [4], including either stenosis or regurgitation, and often mixed. Valvular stenosis without major regurgitation can be treated with simple balloon dilation, and percutaneous PVR is also a good option for patients with suitable RV outflow tract anatomy, while surgical PVR is the most invasive, but reliable treatment. We can choose the best option for individual patients based on the accurate evaluation of the morphology of the PV and etiology of the valve malfunction. A morphological assessment of the PV with ICE would provide useful information in this setting. For percutaneous balloon dilation of the stenotic PV, it is very important to open the valve properly with sufficient relief of the stenosis without a major increase in PR. Close monitoring using ICE during the procedure will be helpful for providing optimal treatment. Recently, percutaneous PVR has been indicated for specific patients, including some with native pulmonary valve failure [13]. It has been reported that ICE is also useful during percutaneous PVR [14,15]. It would be useful not only during treatment but also for evaluation in the postoperative remote period. 

ICE is a relatively invasive modality. However, we could assess the PV and PR with ICE from the internal jugular vein without any complications in our patients. The RV is dilated and/or hypertrophied in many patients with repaired TOF. In addition, the RV outflow tract is often repaired with an outflow patch and the possibility of severe complications (e.g., ventricular perforation) is expected to be relatively low. On the other hand, previous reports on the performance of ICE from the internal jugular vein are scarce [16]. It has been reported that good imaging was obtained by performing ICE via a femoral vein approach as a guide for percutaneous PVR [17]. However, the femoral vein is often occluded due to repeated cardiac catheterization after surgery in patients with congenital heart disease [18]. ICE can be safely used by the internal jugular approach, and it could be a key imaging modality for these patients. 

The present study was associated with several limitations. First, our study enrolled a relatively small number of adult patients with TOF. The usefulness and safety of ICE should be examined in larger study populations and in patients with heart disease other than TOF. The utility and safety for pediatric patients should also be investigated. Second, we did not perform 3D TEE, that is one of the possible modalities to visualize the pulmonary valve. In addition, we did not use quantitative assessment using PISA, which may provide a more precise evaluation of the severity of PR. Further studies using these modalities and techniques will provide useful information to choose the best imaging option in these patients. Third, ICE was performed by a single operator. The safety and quality of images are highly dependent on the experience of the operator. Although overall risks related to ICE are reported to be low, ICE is known to potentially cause transient arrhythmias and vascular-access related complications. Using biplane right ventriculography as a reference guide is helpful to minimize the risks associated with ICE manipulation. Finally, ICE is not feasible in cases after mechanical tricuspid valve replacement, cases with RA/RV thrombus, and intracardiac tumors. Other modalities should be considered in these patients.

## 5. Conclusions

ICE can safely provide better visualization of the PV and PR than TTE in adults with TOF. It is especially useful in patients after PVR, in whom the information provided by TTE can be quite limited.

## Figures and Tables

**Table 1:** Patient Characteristics.

Total Number of Patients	30
Age (years)	33 ± 14
Sex (Male/Female)	12/18
Height (cm)	160 ± 25
Weight (kg)	54 ± 11
Age at last operation (years)	10 ± 12
Diagnosis	-
Tetralogy of Fallot	25 (83%)
PA/VSD	5 (17%)
Surgical repair	-
Transannular patch repair	20 (67%)
Pulmonary valve replacement	8 (27%)
Rastelli type operation	5 (17%)
Unrepaired	1 (3%)
Rhythm	-
Sinus	27 (90%)
Junctional rhythm	1 (3%)
atrial tachycardia	1 (3%)
atrial fibrillation	1 (3%)
QRS duration (msec)	139 ± 37
Cardiothoracic ratio (%)	55 ± 10
BNP (pg/mL)	102 ± 206

PA/VSD, pulmonary atresia with ventricular septal defect; BNP, brain natriuretic peptide.

**Table 2:** Hemodynamic data.

RAP (mm Hg)	6 ± 3
Systolic RVP (mm Hg)	50 ± 19
Systolic PAP (mm Hg)	34 ± 9
Mean PAP (mm Hg)	14 ± 5
Mean PCWP (mm Hg)	8 ± 3
C.O. (L/min)	5.5 ± 2.3
Qp/Qs	1.3 ± 1.1

RAP, right atrial pressure; RVP, right ventricular pressure; PAP, pulmonary arterial pressure; PCWP, pulmonary capillary wedge pressure; C.O., Cardiac output; Qp/Qs, pulmonary to systemic blood flow ratio.

**Table 3:** Transthoracic echocardiographic data.

LVDd (mm)	44 ± 7
LVDs (mm)	29 ± 7
LVEF (%)	64 ± 10
PR grade	-
mild	8 (26%)
moderate	9 (30%)
severe	13 (43%)
TRPG (mm Hg) ^a^	40 ± 17
TPPG (mm Hg) ^b^	25 ± 15

^a^ Three patients in whom TRPG was not recorded were excluded (*n* = 27). ^b^ Three patients in whom TPPG was not recorded were excluded (*n* = 27). LVDd, left ventricular end-diastolic diameter; LVDs, left ventricular end-systolic diameter; LVEF, left ventricular ejection fraction; PR, pulmonary regurgitation; TRPG, transtricuspid pressure gradient; TPPG, transpulmonary pressure gradient.

**Table 4:** Cardiac magnetic resonance imaging.

RVEDV (mL)	269 ± 129
RVEDVI (mL/m^2^)	172 ± 78
RVESV (mL)	167 ± 111
RVESVI (mL/m^2^)	107 ± 69
RVEF (%)	40 ± 9
PRF (%)	37 ± 21
LVEDV (mL)	144 ± 59
LVEDVI (mL/m^2^)	93 ± 37
LVESV (mL)	76 ± 45
LVESVI (mL/m^2^)	49 ± 28
LVEF (%)	48 ± 9

MRI, magnetic resonance imaging; RVEDV, right ventricular end-diastolic volume; RVEDVI, right ventricular end-diastolic volume index; RVESV, right ventricular end-systolic volume; RVESVI, right ventricular end-systolic volume index; RVEF, right ventricular ejection fraction; PRF, pulmonary regurgitation fraction; LVEDV, left ventricular end-diastolic volume; LVEDVI, left ventricular end-diastolic volume index; LVESV, left ventricular end-systolic volume; LVESVI, left ventricular end-systolic volume index; LVEF, left ventricular ejection fraction.

## Data Availability

The data presented in this study are available upon request from the corresponding author.
